# Epidemiology and risk factors for typhoid fever in Central Division, Fiji, 2014–2017: A case-control study

**DOI:** 10.1371/journal.pntd.0006571

**Published:** 2018-06-08

**Authors:** Namrata Prasad, Aaron P. Jenkins, Lanieta Naucukidi, Varanisese Rosa, Aalisha Sahu-Khan, Mike Kama, Kylie M. Jenkins, Adam W. J. Jenney, Susan J. Jack, Debasish Saha, Pierre Horwitz, Stacy D. Jupiter, Richard A. Strugnell, E. Kim Mulholland, John A. Crump

**Affiliations:** 1 Centre for International Health, Dunedin School of Medicine, University of Otago, Dunedin, New Zealand; 2 School of Science, Edith Cowan University, Joondalup, Australia; 3 School of Public Health, University of Sydney, Sydney, Australia; 4 Fiji Centre for Communicable Disease Control, Fiji Ministry of Health, Suva, Fiji; 5 Fiji Health Sector Support Program, Suva, Fiji; 6 Telethon Kids Institute, Perth, Western Australia; 7 Murdoch Childrens Research Institute, Melbourne, Victoria, Australia; 8 College of Medicine, Nursing and Health Sciences, Fiji National University, Suva, Fiji; 9 Wildlife Conservation Society, Melanesia Regional Program, Suva, Fiji; 10 Department of Microbiology and Immunology, University of Melbourne, Victoria, Australia; Liverpool School of Tropical Medicine, UNITED KINGDOM

## Abstract

**Background:**

Typhoid fever is endemic in Fiji, with high reported annual incidence. We sought to identify the sources and modes of transmission of typhoid fever in Fiji with the aim to inform disease control.

**Methodology/Principal findings:**

We identified and surveyed patients with blood culture-confirmed typhoid fever from January 2014 through January 2017. For each typhoid fever case we matched two controls by age interval, gender, ethnicity, and residential area. Univariable and multivariable analysis were used to evaluate associations between exposures and risk for typhoid fever. We enrolled 175 patients with typhoid fever and 349 controls. Of the cases, the median (range) age was 29 (2–67) years, 86 (49%) were male, and 84 (48%) lived in a rural area. On multivariable analysis, interrupted water availability (odds ratio [OR] = 2.17; 95% confidence interval [CI] 1.18–4.00), drinking surface water in the last 2 weeks (OR = 3.61; 95% CI 1.44–9.06), eating unwashed produce (OR = 2.69; 95% CI 1.48–4.91), and having an unimproved or damaged sanitation facility (OR = 4.30; 95% CI 1.14–16.21) were significantly associated with typhoid fever. Frequent handwashing after defecating (OR = 0.57; 95% CI 0.35–0.93) and using soap for handwashing (OR = 0.61; 95% CI 0.37–0.95) were independently associated with a lower odds of typhoid fever.

**Conclusions:**

Poor sanitation facilities appear to be a major source of *Salmonella* Typhi in Fiji, with transmission by drinking contaminated surface water and consuming unwashed produce. Improved sanitation facilities and protection of surface water sources and produce from contamination by human feces are likely to contribute to typhoid control in Fiji.

## Author summary

Modeling suggests that Oceania has surpassed Asia and sub-Saharan Africa as the region with the highest typhoid fever incidence. While Pacific Islands are often neglected due to small population sizes, there is an urgent need to understand the epidemiology of typhoid fever in the region. Fiji, an upper-middle income country in Oceania, has reported an increase in typhoid fever notifications over the last decade. However, the epidemiology of typhoid fever in Fiji is incompletely understood due to gaps in surveillance and lack of epidemiological research on local risk factors. We conducted a case-control study in the Central Division of Fiji to help inform prevention and control strategies. We found unimproved sanitation facilities to be major source of typhoid fever in Fiji, with transmission by drinking contaminated surface water and consumption of unwashed produce. We also found an association between poor water availability and poor hygiene with typhoid fever. Improvements in sanitation facilities to protect surface water and produce from contamination are likely to contribute to improved typhoid control in Fiji. Because of the distinct socio-demographic and environmental conditions found in Oceania, our findings may reflect sources and modes of transmission predominant elsewhere in the region.

## Introduction

Typhoid fever remains a substantial cause of morbidity and mortality in many low- and middle-income countries, with an estimated 17.8 million new episodes annually [1]. By 2015, Oceania had fallen behind both Asia and sub-Saharan Africa to become the region with the lowest coverage of improved drinking water and improved sanitation [2, 3]. Pacific island nations including Fiji [4], Nauru [5], and Papua New Guinea [6] report high case counts of typhoid fever and frequent large outbreaks of the disease. Despite this apparent high incidence, studies to investigate sources and modes of transmission of typhoid fever in the Pacific, where unique socio-demographic, behavioral, and environmental conditions may exist, have been rare [4, 7]. A detailed understanding of local risk factors for typhoid fever is necessary to inform non-vaccine control measures. Furthermore, a robust understanding of the epidemiology of typhoid fever in Fiji is needed to inform decisions about the introduction of recently recommended [8] and prequalified typhoid conjugate vaccine.

Typhoid fever is endemic in Fiji with disease occurring among both rural and urban residents [4, 7]. Blood culture-confirmed typhoid fever infections have increased since the 1990s, rising rapidly since 2005 [9]. By 2010, the incidence of typhoid fever in Fiji, identified by passive surveillance, was 52 per 100 000 population [10]. However, given the limited sensitivity of blood cultures, patterns of health seeking behavior among Fijians, and low access to blood cultures services, the actual incidence of typhoid fever in Fiji is likely to exceed this rate [10].

Previous typhoid fever case-control studies in endemic countries have demonstrated considerable variation in major sources and modes of transmission by location [11, 12].This variation underscores the importance of local research, especially in distinct environments such as those that exist in the smaller island states of Oceania. To identify risk factors for typhoid fever relevant to the distinctive island ecology of Oceania, we conducted a case-control study to inform typhoid control efforts in Fiji’s Central Division.

## Methods

### Setting

The Republic of Fiji, located in the southern Pacific Ocean, consists of 332 islands. Our study was undertaken in the Central Division with a population >370,000 people, representing 43% of the national population, and including the capital city Suva. At the time of the study, Central Division residents were comprised of 56% iTaukei (indigenous Fijians), 38% Fijians of Indian descent, and 6% who identified as being of another ethnicity [13]. The majority of Central Division’s population resided in Suva, and the remainder lived in small rural villages and settlements near major waterways [7]. All public health services in Fiji are provided at the divisional and sub-divisional level. The Colonial War Memorial Hospital (CWMH) in Suva is the largest public referral hospital in the country providing clinical and laboratory services to all of the Central Division.

### Design

We conducted a neighborhood, ethnicity, and age interval matched case-control study from 27 January 2014 through 31 January 2017 in Central Division, Fiji. Cases were defined as patients residing in Central Division who sought care at any Central Division public health facility, and had *Salmonella enterica* serovar Typhi (*Salmonella* Typhi) isolated from blood culture. Those aged >18 years were eligible for enrollment until 1 May 2014 when regulatory approval was received to extend enrollment to all ages. For persons aged <12 years, we interviewed the parent or guardian of the patient. If more than one case was detected in a household, we only enrolled the first case.

Two neighborhood controls were selected for each case, one from a near neighborhood and the other from a more distant neighborhood from where the case arose. We sought near-neighborhood controls 100 paces in a random direction from the case household using a pen spin method. We sought distant-neighborhood controls in the same random direction as a near-neighborhood control, from the next closest river basin in rural areas and from the next closest sub-division in urban and peri-urban areas. We sought potential age-matched controls in the intervals <4 years, 5–14 years, 15–24 years, 25–34 years, 35–44 years, 45–54 years, 55–64 years, 65–74 years, and >75 years of age. We excluded potential controls that had experienced fever within the past one month.

### Data collection

We administered a standardized questionnaire to all participants. The questionnaire sought information on basic demographics and focused on modifiable typhoid fever risk factors and exposures occurring during the two-week period prior to the onset of symptoms for cases and prior to the date of recruitment for controls. Domains of questions included those related to water and food consumption, including kava, a local drink of Fiji made of water infused with *Piper methysticum*, and behavioral practices such as attendance at community gatherings and handwashing. We investigated longer-term environmental factors including the occurrence of floods, droughts, and tropical storms two months prior to the onset of symptoms or interview. We recorded observations on the type and condition of water source and sanitation facility within the bounds of the household and also on distal environmental conditions.

### Laboratory evaluations

Blood cultures were collected from febrile patients at the clinicians’ discretion [14]. Blood was collected in BacT/ALERT standard aerobic and anaerobic blood culture bottles for adults (5–10mL) and pediatric FAN bottles for children (2–5 mL) (bioMérieux, Marcy L’Etoile, France). Bottles were incubated for 5–7 days at 35°C in the BacT/Alert system. Broth from positive blood culture bottles was subcultured on blood, chocolate, and MacConkey agar. Non-lactose fermenting colonies were identified biochemically as probable *Salmonella* Typhi using Microbact identification system, Triple Sugar Iron (TSI), and Lysine Indole Motility (LIM) media. *Salmonella* Typhi were confirmed by slide agglutination, using antibody reagents specific for serogroup O9 and Vi (Difco *Salmonella* Antiserum, Becton Dickinson, Franklin Lakes, NJ USA).

### Statistical analysis

A three-level socioeconomic status (SES) index was created by principal component analysis of education, employment, household conditions, and asset variables [15]. Categorical variables were created for water source and sanitation facilities, with improved municipal water source and undamaged, improved municipal sewerage used as the referent category for each variable. Handwashing behavior questions at pre-specified times (before eating, before cooking, and after defecation) were recoded as an ordinal variable ranging from 0 (never) to 2 (always). A knowledge-based dimension reduction strategy, which considered the causal pathway through directed acyclic graphs, was used to guide variable selection for model building and interpretation of multivariable analysis [16]. Odds ratios (OR) with exact 95% confidence interval (CI) were measured in a univariable analysis using conditional logistic regression for the selected variables. A multivariable conditional logistic regression model with all variables with a p-value <0.1 in the univariable analysis was applied and variables with p-value >0.05 were removed from the model in a step-wise manner. Population attributable fractions (PAF) for categorical, potentially modifiable risk factors were estimated from the prevalence of exposure in case patients and adjusted OR from the multivariable conditional logistic regression model [17, 18]. The proportion of participants with missing data for variables selected for model building ranged from 0–4%. Missing values were imputed using all available data for each participant. Specifically, five complete datasets were created using the multivariate imputation by chained equations (MICE) method of imputation in STATA [19]. Results obtained from imputed datasets were not significantly different from non-imputed data, hence we chose to present our unimputed results. Analysis was done comparing cases to near-neighborhood and distant-neighborhood controls separately and together. Matched ORs with control groups separately were not significantly different to ORs with controls together. Therefore, we chose to present results for the combined control groups. Details on sample size estimation (S1 File), questionnaire used for response collection (S2 File), directed acyclic graph (S1 Fig), and univariable results with analysis of control groups separately (S1 and S2 Tables) are provided in the supporting information. Analyses were conducted with Stata/SE 14.0 for Windows (Stata, TX, USA).

### Research ethics

Ethics approvals were obtained from the Fiji National Health Research Committee, the Human Ethics Committee of the University of Otago, and the Human Research Ethics Committee of Edith Cowan University. Verbal and written details of the study were provided in Fijian, Hindi, or English as necessary and written informed consent was obtained from all participants or their guardians.

## Results

**Fig 1:**
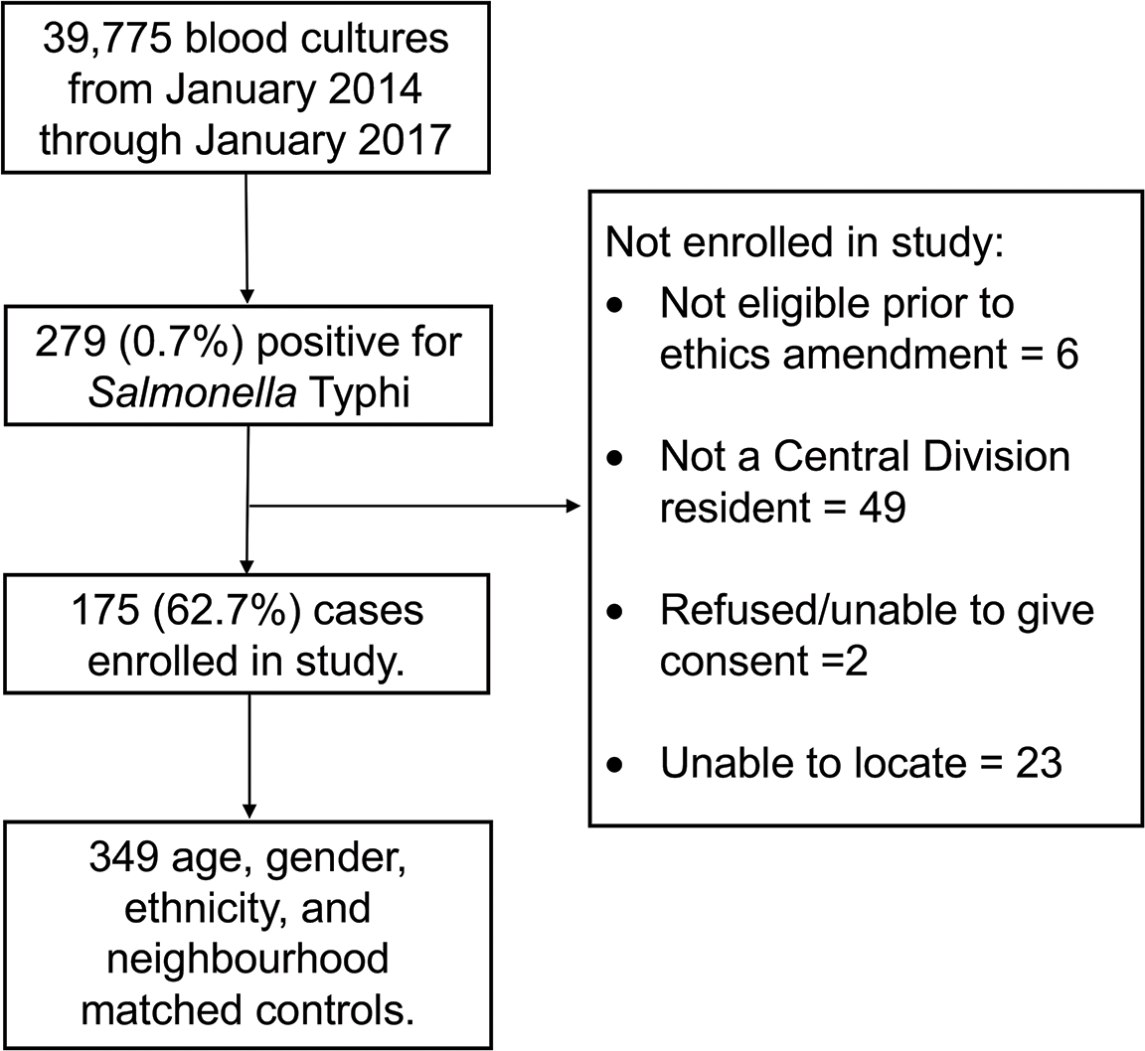
Flowchart illustrating recruitment of patients with Salmonella Typhi infection in Central Division Fiji, 2014–2017.

Of 39,775 blood cultures performed, 279 (0.7%) patients with blood culture-confirmed typhoid fever were identified. No *Salmonella* Paratyphi A, B, or C were isolated during the study period. Of those blood culture-confirmed typhoid fever cases, 175 (62.7%) were enrolled in the case-control study. Reasons for non-enrolment are shown in Fig 1. A total of 349 controls were enrolled in the study. Baseline characteristics of enrolled cases and controls are described in Table 1. Of enrolled cases 84 (48.0%) were from a rural area, while 59 (33.7%) and 32 (18.3%) were from urban and peri-urban areas respectively.

**Table 1:** Baseline characteristics of 175 typhoid fever cases and 349 age, ethnicity, and neighborhood matched controls enrolled in study, Central Division, Fiji, 2014–2017.

Characteristic	Cases		Controls	
	(n = 175)		(n = 349)	
	N	(%)	N	(%)
**Total**	175	(100.0)	349	(100.0)
**Gender**				
Male	86	(49.1)	171	(49.0)
**Age group**				
< 5 years	9	(5.1)	15	(4.3)
5–17 years	44	(25.1)	84	(24.1)
18–49 years	97	(55.4)	207	(59.3)
≥ 50 years	25	(14.3)	43	(12.3)
**Ethnicity**				
iTaukei (indigenous Fijians)	166	(94.9)	331	(94.8)
Fijians of Indian descent	9	(5.1)	18	(4.9)
**Residential Area**				
Urban	59	(33.7)	118	(33.5)
Peri-urban	32	(18.3)	63	(18.1)
Rural area	84	(48.0)	168	(47.9)

In the univariable analysis (Table 2), the odds of a case being from a low SES index household was almost 3 times that of controls (matched OR 2.98 and 95% CI 1.64–5.44). Cases more frequently accessed their main water source from outside the house (OR = 2.96, 95% CI 1.20–7.29), had interrupted water availability (OR = 2.40, 95% CI 1.39–4.12), and drank water from an untreated source in the last two weeks (OR = 1.80, 95% CI 1.07–3.03). Among participants who reported drinking water from a source other than their main household water source, cases were more likely to have drunk water from a surface water source than controls (OR = 3.04, 95% CI 1.33–6.92). No difference in kava consumption was observed between cases and controls.

**Table 2:** Univariable analysis of risk factors for blood-culture-confirmed typhoid fever among 175 cases and 349 age, ethnicity, and neighborhood matched controls, Central Division, Fiji, 2014–2017.

Risk factor/ Exposure	Number and (%) of cases with risk factor/ exposure		Number and (%) of controls with risk factor/ exposure		Conditional odds ratio	Exact 95% confidence Intervals	p-value
	Total no. of cases = 175		Total no. of controls = 349				
	N	(%)	N	(%)			
**Household**							
High socioeconomic status index	32	(18.3)	98	(28.1)	ref		
Medium socioeconomic status index	57	(32.6)	127	(36.4)	1.72	0.97–3.06	0.063
Low socioeconomic status index	86	(49.1)	124	(35.5)	2.98	1.64–5.44	<0.001
Animals in household	86	(49.1)	195	(55.9)	0.72	0.49–1.06	0.099
**Water source, treatment, and drinking**							
Main household water source							
Piped treated	88	(50.3)	172	(49.3)	ref		
Piped untreated	31	(17.7)	77	(22.1)	0.50	0.18–1.42	0.196
Rain water	4	(2.3)	5	(1.4)	1.67	0.38–7.39	0.502
Surface water	52	(29.7)	95	(27.2)	1.28	0.35–4.70	0.713
Main water source accessed from outside house	118	(67.4)	68	(19.5)	2.96	1.20–7.29	0.018
Water not always available from main source	49	(28.0)	62	(17.8)	2.40	1.39–4.12	0.002
Treated water in house	50	(28.6)	107	(30.7)	0.89	0.57–1.39	0.596
Stored water in house	139	(79.4)	269	(77.1)	1.16	0.73–1.85	0.530
Drank untreated water	76	(43.4)	126	(36.1)	1.80	1.07–3.03	0.027
Only drank water from main household water source	104	(59.4)	232	(66.5)	ref	-	
Drank from an alternate water source (non-surface water source)	53	(30.3)	100	(28.7)	1.31	0.78–2.21	0.313
Drank from an alternate water source (surface water source)	18	(10.3)	17	(4.9)	3.04	1.33–6.92	0.008
Drank water at a mass gathering	14	(8.0)	19	(5.4)	1.51	0.74–3.10	0.256
Consumed ice	69	(39.4)	131	(37.5)	1.14	0.77–1.68	0.524
Drank water/other drink from a street vendor	57	(32.6)	97	(27.8)	1.34	0.86–2.09	0.189
Drank kava ^a^	71	(40.6)	164	(47.0)	0.68	0.43–1.06	0.091
**Food and behavior**							
Did not wash produce before eating	53	(30.3)	48	(13.8)	3.48	2.06–5.89	<0.001
Stored food	121	(69.1)	252	(72.2)	0.83	0.53–1.29	0.399
Shared food on the same plate	20	(11.4)	22	(6.3)	2.13	1.07–4.25	0.032
Ate outside of house	77	(44.0)	121	(34.7)	1.56	1.04–2.34	0.032
Consumed dairy products	156	(89.1)	316	(90.5)	0.81	0.41–1.62	0.551
Ate kai/mussels	72	(41.1)	181	(51.9)	0.57	0.37–0.87	0.009
Ate lolo/coconut milk	129	(73.7)	276	(79.1)	0.61	0.36–1.06	0.080
Attended a mass gathering	64	(36.6)	97	(27.8)	1.59	1.05–2.40	0.029
**Sanitation and hygiene**							
Shared toilet with non-household members	24	(13.7)	42	(12.0)	1.30	0.64–2.67	0.461
Householders built their own toilet	92	(52.6)	154	(44.1)	1.53	1.12–2.30	0.039
Have a unimproved/damaged improved sewerage system ^b^	167	(95.4)	318	(91.1)	2.80	1.02–7.65	0.045
Undamaged improved, municipal sewerage	8	(4.6)	31	(8.9)	ref	-	
Unimproved pit latrine	16	(9.1)	10	(2.9)	26.62	5.20–136.37	<0.001
No toilet/open defecation	5	(2.9)	3	(0.9)	14.17	1.97–102.14	0.009
Damaged improved, municipal sewerage	7	(4.0)	10	(2.9)	5.28	1.23–22.73	0.025
Improved pit latrine	62	(35.4)	109	(31.2)	4.40	1.46–13.26	0.009
Intact septic	77	(44.0)	186	(53.3)	2.41	0.86–6.71	0.093
Separate water source for washing hands	35	(20.0)	90	(25.8)	0.64	0.38–1.08	0.095
High handwashing frequency after defecation					0.41	0.27–0.62	<0.001
Used soap for handwashing	80	(45.7)	227	(65.0)	0.38	0.24–0.58	<0.001
**Environment**							
Heavy to moderate rain 2 weeks	87	(49.7)	169	(48.4)	1.09	0.69–1.72	0.700
Heavy to moderate rain 2 months	98	(56.0)	180	(51.6)	1.31	0.83–2.09	0.245
Household evacuated 2 weeks	2	(1.1)	6	(1.7)	0.59	0.10–3.54	0.567
Household evacuated 2 months	3	(1.7)	7	(2.0)	0.50	0.03–7.99	0.624
Drought 2 weeks	1	(0.6)	1	(0.3)	2.00	0.13–31.98	0.624
Drought 2 months	1	(0.6)	3	(0.9)	0.59	0.05–7.43	0.685
Flooding adjacent 2 weeks	5	(2.9)	12	(3.4)	0.79	0.24–2.56	0.695
Flooding adjacent 2 months	2	(1.1)	8	(2.3)	0.47	0.09–2.34	0.356
Village flooded 2 weeks	6	(3.4)	7	(2.0)	1.92	0.57–6.52	0.294
Village flooded 2 months	5	(2.9)	8	(2.3)	1.31	0.38–4.45	0.671
Toilet flooded 2 weeks	2	(1.1)	1	(0.3)	4.00	0.36–44.1	0.258
Toilet flooded 2 months	1	(0.6)	1	(0.3)	2.00	0.13–31.98	0.624
River/stream flooded 2 weeks	14	(8.0)	16	(4.6)	2.24	0.90–5.60	0.083
River/stream flooded 2 months	19	(10.9)	19	(5.4)	2.42	1.13–5.12	0.020
Farms above water collection	11	(6.3)	13	(3.7)	2.38	0.82–6.88	0.109
Livestock above water collection	7	(4.0)	5	(1.4)	3.78	0.95–15.4	0.059
Logging above river basin	1	(0.6)	4	(1.1)	0.43	0.04–4.61	0.489
Road building above river basin	3	(1.7)	11	(3.2)	0.21	0.02–2.01	0.179
Dams above river basin	71	(40.6)	112	(32.1)	2.18	1.22–3.91	0.009

Odds ratios were estimated using conditional logistic regression. All exposures are focused on the 2-week period prior to onset of symptoms for cases and the date of recruitment for controls, unless specified otherwise.

^a^ Traditional Fijian drink (water infused with *Piper methysticum*).

^b^ Summary variable of all sanitation facilities.

Compared to controls, cases were more likely to eat unwashed produce (OR = 3.48, 95% CI 2.06–5.89). Cases were also more likely to have eaten outside of the house in the past two weeks (OR = 1.56, 95% CI 1.04–2.34), and have attended a mass gathering in the past two weeks (OR = 1.59, 95% CI 1.05–2.40).

When categorized by latrine type, cases were more likely to have either an unimproved pit latrine (OR = 26.62, 95% CI 5.20–136.37), or no toilet or latrine in the household (OR = 14.17, 95% CI 1.97–102.14), or a damaged, improved municipal sewerage (OR = 5.28, 95% CI 1.23–22.73), or an improved pit latrine (OR = 4.40, 95% CI 1.46–13.26) compared to controls. Furthermore, cases were more likely than controls to report having a latrine built by someone within their own household (OR = 1.53, 95% CI 1.12–2.30). With respect to hygiene behaviors, a higher handwashing score after defecating (OR = 0.41, 95% CI 0.27–0.62) and using soap for handwashing (OR = 0.38, 95% CI 0.24–0.58) were associated with lower odds of typhoid fever.

In terms of distal environmental factors, compared to controls, cases were more likely to have experienced flooding of their nearest river or stream in the past two months (OR = 2.42, 95% CI 1.13–5.12). Cases were also more likely to have a dam located upstream from their closest river basin (OR = 2.18, 95% CI 1.22–3.91) than controls.

On multivariable analysis ten exposures remained independently associated with typhoid fever (Table 3) including drinking water from an alternative surface water source in the last two weeks (OR = 3.61, 95% CI 1.44–9.06), not having constant water availability (OR = 2.17, 95% CI 1.18–4.00), and eating unwashed produce (OR = 2.69, 95% CI 1.48–4.91). The population attributable fraction (PAF) for these exposures was 7%, 15%, and 19%, respectively. Having any unimproved sewerage system or a damaged improved sewerage system was associated with increased odds of typhoid fever (OR = 4.30, 95% CI 1.14–16.21). The summary PAF for these poor sanitation facilities was 72%. Frequent handwashing after defecating was associated with lower odds of *Salmonella* Typhi infection (OR = 0.57; 95% CI 0.35–0.93), as was using soap for handwashing (OR = 0.61, 95% CI 0.37–0.95).

**Table 3:** Multivariable analysis of risk factors for blood-culture-confirmed typhoid fever among 175 cases and 349 age, ethnicity, and neighborhood matched controls, Central Division, Fiji, 2014–2017.

Risk factor/ Exposure	Conditional odds ratio	95% confidence intervals	P-value	Population attributable risk
Drank from an alternate water source (surface water source)	3.61	1.44–9.06	0.006	0.07
Water not always available from main source	2.17	1.18–4.00	0.013	0.15
Did not wash produce before eating	2.69	1.48–4.91	0.001	0.19
Had any unimproved sewerage/damaged improved sewerage system ^a^	4.30	1.14–16.21	0.031	0.72
Undamaged, improved, municipal sewerage	ref	-		
Unimproved pit latrine	49.47	9.42–259.92	0.000	
No toilet/Open defecation	9.87	0.85–114.35	0.067	
Damaged improved, municipal sewerage	6.00	1.22–29.51	0.027	
Improved pit latrine	5.55	1.46–21.09	0.012	
Intact septic	3.73	1.08–13.81	0.049	
High handwashing frequency after defecation	0.57	0.35–0.93	0.025	
Use soap for handwashing	0.61	0.37–0.95	0.041	-0.23

Odds ratios were estimated using conditional logistic regression. All exposures are focused on the 2-week period prior to onset of symptoms for cases and the date of recruitment for controls, unless specified otherwise.

^a^ Summary variable of all sanitation facilities.

## Discussion

To our knowledge, this is the first case-control study to investigate sources and modes of transmission for typhoid fever in Fiji. We demonstrate that unimproved sanitation facilities are a likely major source of *Salmonella* Typhi, with transmission occurring through drinking contaminated surface water and eating unwashed produce. We also show typhoid to be common among both rural and urban populations in Fiji (Table 1). Improving sanitation facilities and increasing access to safe water and clean produce for rural populations presents a major challenge in Fiji and more broadly for other island nations of Oceania.

The median age of cases in our study was 29 years, which is higher than the median age reported in several other typhoid endemic areas [11, 12]. While this may suggest a distinct typhoid epidemiology in Fiji, under-ascertainment by blood culture of typhoid fever among younger age groups is also a possibility as younger patients in Fiji have been reported to receive early empiric treatment for fever without blood culture [4]. Of cases in our study, 95% were among iTaukei. This finding is in contrast to a sero-epidemiologic survey conducted in Fiji in 2013, which found both iTaukei and Fijians of Indian ancestry to have similar sero-prevalence of antibodies against the Vi antigen of *Salmonella* Typhi [20]. Besides Vi serology being an inaccurate measure of *Salmonella* Typhi infection, a possible explanation for this difference is under-ascertainment by blood culture of typhoid fever among Fijians of Indian ancestry, who have been shown to preferentially present to private general practitioners and are again likely to receive early and empiric treatment for fever without blood culture [21].

Our results suggest that unimproved or damaged sanitation is a major source of *Salmonella* Typhi in Fiji. We found that people without access to improved sanitation facilities or with damaged improved sewerage systems were at particular risk. Those with typhoid fever were more likely than controls to have someone within their household build their toilet (Table 2). Others have shown latrines built by persons without expertise to be poorly constructed, built into permeable soil, and subject to flooding [7, 22]. Notably, households with improved pit latrines had greater odds of typhoid fever in our study, compared to undamaged, improved municipal sanitation (Table 3). In Fiji, a common ‘improvement’ is use of buried steel drums as the receptacle for sewerage [23]. Such receptacles are subject to flooding, corrosion, and leakage [23] leading to contamination of surface water and crops by human feces [24]. Interestingly, eating unwashed produce was an independent risk factor for typhoid in our study. Related research has shown that gardens of patients with typhoid fever were more often positioned closer to the household toilet or septic tank than in control households and the majority of cases propagated vegetables directly on or below the toilet drainage area [25]. In contrast to water-related factors, poor sanitation has seldom been reported as a risk factor for typhoid fever [11, 12, 26], highlighting the value of our local research.

Contaminated drinking water is commonly identified as a risk factor for typhoid fever in case-control studies [26]. We showed that cases were more likely to report having poor water availability than controls (Table 3). Intermittent access to water is a common problem for households in many low-resource countries [27] and can lead to increased risk of typhoid fever by a number of mechanisms. First, in Fiji, as in many other locations, poor water availability from a primary source results in households shifting periodically to alternative water sources, including unsafe surface water [28, 29]. Second, during periods of reduced water supply, households may rely on stored drinking water that is not disinfected. Although we did not demonstrate consumption of stored water as a risk factor for typhoid fever in Fiji, related research showed a significantly higher concentration of *E*. *coli* in stored drinking water in typhoid case households compared to control households, but not in source water [25]. Third, pressure drops associated with regular interruption in reticulated water supplies can result in negative pressure situations which, when combined with leaks in the distribution system, can result in inflow of environmental material [30]. While our study was not designed to confirm this mechanism, we hypothesize that this may occur in Fiji and warrants further investigation. Finally, poor water availability can affect the quality of sanitation facilities that rely on water as well as affect personal hygiene [31, 32]. However, our multivariable analysis showed poor water availability as associated with increased odds of having typhoid fever even after adjustment for unimproved sanitation and handwashing frequency.

Factors related to hygiene were also independently associated with typhoid in our study setting (Table 3). Frequent handwashing after defecating and using soap for handwashing were associated with lower odds of typhoid fever. Both of these handwashing behaviors have been associated with reduced risk of typhoid in other studies [12, 28].

Distal conditions within the water catchment have yet to be thoroughly evaluated in case-control studies of typhoid fever. However, geospatial studies are beginning to examine such relationships on broad spatial scales [7, 33, 34]. Although no distal environmental conditions were statistically associated with typhoid fever in our multivariable model, experiencing flooding of the nearest river or stream in the past two months and reporting dams upstream in the river basin were significant in univariable analysis. Descriptive accounts of dam construction and bursts have been linked to an increase in typhoid cases in Nigeria [35]. However, the basis for these risks is not well understood and could be the subject of future research. At a sub-catchment scale, typhoid infection and disease in the Fijian setting has been linked with forest fragmentation, increased erosion, rainfall, and flood risk [7, 34]. It is likely that such environmental factors result in overflow and damage to already poor sanitation facilities leading to contamination of produce and drinking water sources.

Our study had a number of limitations. First, recall bias may influence the reliability of potential exposures over the long incubation period of typhoid fever [36] and social desirability bias is a common concern for sanitation and hygiene questions [37]. We sought to control the former by making observations of sanitation facilities. However, observations and more objective measures of handwashing practices were not possible. Therefore, our findings on handwashing should be interpreted with caution. Second, since the interviewers in this study knew the case status of the participant, it is possible that they may have acquired differential exposure information from cases. However, our questionnaire was standardized and interviewers were trained to ensure cases and controls were questioned in the same way. Third, the relative homogeneity of sampled environments and the collinear relationship of many factors may have masked the detection of potential risk factors. However, by recruiting a second control in a distant neighborhood and by obtaining a large sample size we expected to address power concerns of ‘over-matching’ and have adequate statistical power to identify minor associations. Finally, since case detection was by passive surveillance at public healthcare facilities, we are unlikely to have identified all typhoid fever illnesses and we may have missed cases that were more likely to be treated empirically or preferentially access private healthcare services. This could have also resulted in us missing exposures associated with increased risk of typhoid fever in these populations. Future research should include case finding at private health care facilities.

In conclusion, our study demonstrates that unimproved and damaged sanitation facilities are an important source of *Salmonella* Typhi in Fiji. Transmission appears to be by drinking contaminated surface water and consumption of unwashed produce, and is common in both rural and urban populations. Poor hygiene practices also appear to increase odds for typhoid fever. Although not detected in this study, landscape factors contributing to flooding of poor sanitation facilities may also contribute to enhanced risk [7, 34]. The situation in Fiji may reflect sources and modes of transmission predominant elsewhere in Oceania, where typhoid incidence is also high [1] and similar socio-demographic and environmental circumstances prevail. Meeting the 2030 Sustainable Development Goals goals [2] to improve sanitation facilities and protect surface water and produce from contamination by human feces are likely to contribute to typhoid control in Fiji. Central or household based water disinfection would also help to render fecally contaminated water safe for consumption. Such long-term socioeconomic, land and water management, and sanitation infrastructure developments together with uptake of typhoid conjugate vaccination in the interim are likely to result in effective typhoid control in Fiji and Oceania.

## Supporting information

S1 File Sample size estimation. (DOCX)

S2 File Study questionnaire. (DOCX)

S3 File STROBE checklist. (DOC)

S1 Fig Directed acyclic graph used to guide variable selection. (DOCX)

S1 Table Univariable analysis of risk factors for blood-culture-confirmed Salmonella Typhi among 175 cases and 175 age, ethnicity, and near-neighborhood matched controls, Central Division, Fiji, 2014–2017. (DOCX)

S2 Table Univariable analysis of risk factors for blood-culture-confirmed Salmonella Typhi among 175 cases and 175 age, ethnicity, and distant-neighborhood matched controls, Central Division, Fiji, 2014–2017. (DOCX)
