# Supplementary material for: Epidemiology and risk factors for typhoid fever in Central Division, Fiji, 2014–2017: A case-control study
Source: PLoS Negl Trop Dis. 2018 Jun 8;12(6):e0006571. doi: 10.1371/journal.pntd.0006571 (PMC6010302; doi:10.1371/journal.pntd.0006571)
Supplement: S1 File — (DOCX) [file pntd.0006571.s001.docx]

**SI File: Sample size estimation**

We hypothesized that fecally contaminated water is a likely risk factor for transmission of typhoid fever in Fiji. Our sample size estimation was based on a number of key assumptions. Water use practices in rural Fiji are quite homogeneous. Therefore, we assume differences of around 20% is safe water use practices between cases and controls (i.e., roughly 80% of the controls were also exposed to the water contaminated with *Salmonella* Typhi). To achieve 80% power for a two-sided p-value of <0.05, we would require 180 cases and 360 matched controls to establish our hypothesis (see table below).

**Required sample size for achieving 80% power and an expected odd ratio of 2**

| Estimated % exposed among the controls | No of controls/case | Power | Alpha | No of cases | No of matched controls |
| --- | --- | --- | --- | --- | --- |
| 80% | 2 | 80% | 0.05 | 180 | 360 |

Due to limitations in study resources as well as exclusion of cases that did not consent to participate in the study, our final sample size included 175 cases and 349 matched controls. While this was slightly less than the required sample size based on our power calculation, we considered it to provide sufficient statistical power to detect exposures associated with typhoid fever in Fiji.
