# Supplementary material for: Epidemiology and risk factors for typhoid fever in Central Division, Fiji, 2014–2017: A case-control study
Source: PLoS Negl Trop Dis. 2018 Jun 8;12(6):e0006571. doi: 10.1371/journal.pntd.0006571 (PMC6010302; doi:10.1371/journal.pntd.0006571)
Supplement: S2 File — (DOCX) [file pntd.0006571.s002.docx]

**Epidemiology of Typhoid fever in Fiji**

**CRF: Enrolment questionnaire for case and control**

**[Note: You need not to administer the questionnaire, the way it is written in English. Use local terminologies so that it is easily understandable by the interviewee. Always start with an open question and then lead the respondents with closed questions as appropriate. Never prompt an answer; provide interviewee with sufficient time for an appropriate response. There are instructions after certain questions; follow them]**

1. Is it a case or control: [Case=1; Control=2]
2. Please insert the corresponding case or control ID:

**Demographic information:**

1. Subject ID:
2. Date of enrolment:

DD MM YYYY

1. Age: Day/Month/Year

(Use an event calendar to calculate age if exact date of birth is unknown)

1. Date of birth:

DD MM YYYY

(Use event calendar to approximate the date of birth)

1. Sex: [Male=1;Female=2]
2. Residential area: [Urban=1; Rural=2; Peri-urban=3]
3. Insert the GPS coordinate of the resident: S ^o^  .

E ^o^ .

1. What is your (your child’s) primary occupation:

[Dependant (if the subject is less than five years) =1; student=2; manual labourer=3 skilled labourer (obtained through certified course) =4; farmer= 5; business=6; small traders=7; professional (after completing a recognized degree e.g. teacher, doctor, engineer, any office work etc.)=8; housewife=9; Unemployed=10; sedentary=11]

Note: Circle the given answer and also put the code in the box

1. How many people live in your (your child’s) household?

(Confirm by asking who they are)

1. Can you please tell the age and sex of the people live in your (your child’s) household

(Can construct a table to list all the people)

| No. | Relation (mother, father, brother, sister etc.) | Age | Sex[Male=1;Female=2] |
| --- | --- | --- | --- |
| 1 |  |  |  |
| 2 |  |  |  |
| 3 |  |  |  |
| 4 |  |  |  |
| 5 |  |  |  |
| 6 |  |  |  |
| 7 |  |  |  |
| 8 |  |  |  |
| 9 |  |  |  |
| 10 |  |  |  |
| 11 |  |  |  |
| 12 |  |  |  |
| 13 |  |  |  |
| 14 |  |  |  |
| 15 |  |  |  |

1. How many sleeping rooms are there in your (your child’s) household?
2. How far have you (your child) studied in an educational institute?

[Attended primary=1; completed primary=2; secondary=3; completed secondary=4; Post- secondary=5; University=6; No formal education=7; religious education only=8]

Note: Circle the given answer and also put the code in the box

**Family history**

1. Has anybody in your (your child’s) household had a fever in the last two weeks? [Yes=1;No=2] If “NO” go to Q 16 otherwise continue.

15a. if yes do you know whether any one of them was diagnosed as typhoid fever? [Y=1; No=2]

15b. was anyone of them treated with antimicrobials/any medicine in the last two weeks? [Yes=1; No=2]

15c.If yes can you please name the medicine? _____________________________________

1. Do you know anybody in your (your child’s) household has been diagnosed with gall bladder disease? [Yes=1;No=2]
2. Has anyone in your household been told that they are a typhoid carrier? [Yes=1;No=2]

If “No” go to Q 18 otherwise continue

17a. If yes who? _____________________________________________________________

17b. If yes when he/she was told days/months/years ago

17c. if yes have they been treated? [Yes=1; No=2]

1. Have you ever been vaccinated for typhoid fever? [Yes=1;No=2]

18a.If yes when? Days/months/years ago

1. Has anybody in your (your child’s) household been vaccinated for typhoid fever? [Yes=1;No=2]

If yes,

19a. When days/moths/years ago

19b. How many of the household members were vaccinated

**Household asset:**

Note: for question no 19-21 circle the given answer and also put the code in the box

1. What is the predominant structure of your (your child’s) house floor [only one can be ticked]
   1. Earth/sand [Yes=1; No=2]
   2. Dung [Yes=1; No=2]
   3. Wood plunks [Yes=1; No=2]
   4. Palm/bamboo [Yes=1; No=2]
   5. Parquet or polished wood [Yes=1; No=2]
   6. Cement [Yes=1; No=2]
   7. Ceramic tiles [Yes=1; No=2]
   8. Carpet [Yes=1; No=2]
2. Do you have any of the following in your household: [Tick all that apply]
   1. Electricity [Yes=1; No=2]
   2. Television [Yes=1; No=2]
   3. Radio [Yes=1; No=2]
   4. Refrigerator [Yes=1; No=2]
   5. Bi cycle [Yes=1; No=2]
   6. [Yes=1; No=2]
   7. Motorized vehicle (car, bike etc.) [Yes=1; No=2]
   8. Telephone ( land/mobile) [Yes=1; No=2]
   9. Non-motorized vehicle [Yes=1; No=2]
   10. Agricultural land [Yes=1; No=2]
   11. None of the above [if the person interviewed has none of the above put 0]
3. Do any of the following animals live in your household [tick all that apply]
   1. Cow [Yes=1; No=2]
   2. Goat [Yes=1; No=2]
   3. Sheep [Yes=1; No=2]
   4. Horse/donkey/mule [Yes=1; No=2]
   5. Fowl [Yes=1; No=2]
   6. Dog [Yes=1; No=2]
   7. Cat [Yes=1; No=2]
   8. Other [Yes=1; No=2]
   9. No animal [if there’re is no animal in the interviewees house put 0]

**Water Source and treatment**

1. What is the main water source for drinking water in your household [ only one can be ticked]
   1. Piped into the house (from a public supply system) [Yes=1; No=2]
   2. Piped into the yard (from a public supply system) [Yes=1; No=2]
   3. Shared public tap [Yes=1; No=2]
   4. Shallow tube well [Yes=1; No=2]
   5. Deep tube well [Yes=1; No=2]
   6. Covered well [Yes=1; No=2]
   7. Open well [Yes=1; No=2]
   8. River water [Yes=1; No=2]
   9. Lake water [Yes=1; No=2]
   10. Dam [Yes=1; No=2]
   11. Pond [Yes=1; No=2]
   12. Rain water [Yes=1; No=2]
   13. Spring water (protected) [Yes=1; No=2]
   14. Spring water (unprotected) [Yes=1; No=2]
   15. Bottle water ( bought) [Yes=1; No=2]
   16. Other____________ [Yes=1; No=2]

Note: Circle the given answer and also put the code in the box

23a. Is water available all the time from the main source mentioned above?

[Yes-1; No=2] If “YES” go to Q 24 otherwise continue

23b. If ‘no’ how often has the water been available from this main source in the last two weeks?

a. Several hours a day=1

b. Few times in a week=2

c. Never=3

Note: Circle the given answer and also put the code in the box

1. Is water from the main source treated? [Yes=1;No=2;Don’t Know=3]

24a. If yes, is it treated all the time? [Yes=1; No=2; Don’t Know=3]

1. Do you usually treat drinking water at home? [Yes=1;No=2]

If “no” go to question no 26 otherwise continue

25a. If yes what method do you usually use to treat water? [Only one can be ticked]

- 1. Boil=1
  2. Filter through a cloth=2
  3. Filter through ceramic=3
  4. Add chlorine, alum or any tablets=4
  5. Solar disinfection=5

Note: Circle the given answer and also put the code in the box

1. In the last two weeks did you (your child) drink water that was not treated?

[Yes=1; No=2]

1. If yes how many times you (your child) drank untreated water in the last two weeks?
2. In the last two weeks preceding your (your child) present illness did you (your child) drink water at home from any of the following? (Confirm that main source of water and potable water is same). And can you please remember approximately how many times you drank water from the sources you have mentioned? (prompt sources) [Yes=1; No=2]

| Source | Y/N | How many times |
| --- | --- | --- |
| 1. Piped into the house ( from a public supply system) |  |  |
| 1. Piped into the yard ( from a public supply system) |  |  |
| 1. Shared public tap |  |  |
| 1. Shallow tube well |  |  |
| 1. Deep tube well |  |  |
| 1. Covered well 2. Open well |  |  |
| 1. River water |  |  |
| 1. Lake water |  |  |
| 1. Dam |  |  |
| 1. Pond |  |  |
| 1. Rain water |  |  |
| 1. Spring water (protected) |  |  |
| 1. Spring water (unprotected) |  |  |
| 1. Bottle water ( bought) |  |  |
| 1. Other____________ |  |  |

1. Do you store water? [Yes=1; No=2]

If “NO” go to Q30 otherwise continue

29a. If yes where do you store water?

- 1. In the house=1
  2. In the courtyard=2
  3. Overhead tank=3

Note: Circle the given answer and also put the code in the box

29b. Is your storage container (most commonly used)

- 1. Narrow mouthed and capped=1
  2. Narrow mouthed and uncapped=2
  3. Wide mouthed and capped=3
  4. Wide mouthed and uncapped=4

Note: Circle the given answer and also put the code in the box

29c. how do you dispense water from the container?

- 1. Pour=1
  2. Scoop with a cup=2
  3. Scoop with a ladle=3
  4. Through a faucet=4

Note: Circle the given answer and also put the code in the box

1. In the last two weeks preceding your (your child) present illness did you (your child) drink water outside of home from any of the following? And can you please remember how many times you drank water from the sources? (prompt sources) [Yes=1; No=2]

| Source | Y/N | How many times |
| --- | --- | --- |
| 1. Piped into house (from a public supply system) |  |  |
| 1. Piped into the yard ( from a public supply system) |  |  |
| 1. Shared public tap |  |  |
| 1. Shallow tube well |  |  |
| 1. Deep tube well |  |  |
| 1. Covered well 2. Open well |  |  |
| 1. River water |  |  |
| 1. Lake water |  |  |
| 1. Dam |  |  |
| 1. Pond |  |  |
| 1. Rain water |  |  |
| 1. Spring water (protected) |  |  |
| 1. Spring water (unprotected) |  |  |
| 1. Bottle water ( bought) |  |  |
| 1. Other____________ |  |  |

[Continue if answer to this question is YES otherwise go to Q 34]

1. If yes where did you (your child) drink water from the source outside the house?
   1. Work place (mention the place e.g. office/farm/agricultural land etc.)=1
   2. School=2
   3. Restaurant=3
   4. Community gathering (funeral/religious/wedding)=4
   5. Other_________________=5

Note: Circle the given answer and also put the code in the box

1. If yes do you know whether the water you (your child) drank outside was treated? [Yes=1;No=2; Do not know=3]
2. Was the water you (your child) drank outside of home was stored?

[Yes=1; No=2; Do not know=3]

33a. if yes do you know where it was stored?

[Yes=1; No=2; Do not know=3]

33b. if yes can you tell me how it was dispensed

- 1. Pour=1
  2. Scoop with a cup=2
  3. Scoop with a ladle=3
  4. Through a faucet=4
  5. Do not know=5

Note: Circle the given answer and also put the code in the box

1. Did you (your child) use ice in the drinking water or beverages in the last two weeks?

[Yes=1; No=2; Do not know=3]

1. Did you (your child) drink water/beverages in any restaurant in the last two weeks?

[Yes=1; No=2; Do not know=3]

1. Did you (your child) drink any beverages/water from the street vendor?

[Yes=1; No=2; Do not know=3]

1. Do you (your child) drink KAVA? [Yes=1;No=2]

If “NO” go to Q 40 otherwise continue

1. Did you share KAVA with anybody in the last two weeks? [Yes=1; No=2]
2. Do you know the source of water used to prepare Kava?
   1. Piped into the house ( from a public supply system)=1
   2. Piped into the yard ( from a public supply system)=2
   3. Shared public tap=3
   4. Shallow tube well=4
   5. Deep tube well=5
   6. Covered well=6
   7. Open well=7
   8. River water=8
   9. Lake water=9
   10. Dam=10
   11. Pond=11
   12. Rain water=12
   13. Spring water (protected)=13
   14. Spring water (unprotected)=14
   15. Bottle water ( bought)=15
   16. Other____________=16

Note: Circle the given answer and also put the code in the box

**Food:**

1. Do you (your child) grow your own produce? [Yes=1;No=2]

If “NO” go to Q 44 otherwise continue

1. If yes what type of manure/fertilizer do you use to grow those vegetables?
   1. Chemical fertilizer=1
   2. Livestock or poultry manure=2
   3. Other natural fertilizer=3
   4. Human excreta=4
   5. Do not use any manure or fertilizer=5

Note: Circle the given answer and also put the code in the box

1. How much fertilizers do you use per month [skip Q 42&43 if you have circled “e” in Q 41]
   1. 10-100 kg=1
   2. 101-500 kg=2
   3. 501-1000 kg=3
   4. Over 1000 kg=4

Note: Circle the given answer and also put the code in the box

1. How often do you use fertilizers?
   1. Daily=1
   2. Weekly=2
   3. Monthly=3
   4. Other_________________=4

Note: Circle the given answer and also put the code in the box

1. Do you (your child) eat produce? [Yes=1;No=2]
2. Did you (your child) eat produce in the last two weeks? [Yes=1;No=2]
3. Do you (your child) wash produce before eating? [Yes=1;No=2]
4. Did you (your child) eat unwashed produce in the last two weeks? [Yes=1;No=2; Do not know=3]
5. Who usually prepares food in your household?
   1. Self=1
   2. Spouse=2
   3. Parent=3
   4. Grandparent=4
   5. Sibling=5
   6. Other relative=6
   7. Domestic help=7

Note: Circle the given answer and also put the code in the box

1. Can you please state the following of the person who prepares food in your household
   1. Age Years
   2. Gender [Male=1; Female=2]
   3. Any recent history of typhoid fever [Yes=1; No=2; Do not know=3]
   4. Any history of gall bladder disease [Yes=1; No=2; Do not know=3]
2. Do you usually store cooked food for subsequent meals? [Yes=1;No=2]

50a. if yes, do you refrigerate stored food? [Yes=1; No=2]

50b. if yes, do you usually heat pre-cooked food before consumption ` [Yes=1; No=2}

1. Do you (your child) usually eat or share from the same plate with others? [Yes=1;No=2]
2. Did you (your child) eat or share food with others in the last two weeks?

[Yes=1; No=2; Do not know=3]

1. Did you (your child) eat any food from outside of home in the last two weeks (other than places mentioned above)? [Yes=1; No=2; Do not know=3]

55a. If yes where?

- 1. Restaurant=1
  2. Street vendor=2
  3. Friend /relatives house=3
  4. Other_____________=4

Note: Circle the given answer and also put the code in the box

1. Did you (your child) have any of the dairy products in the last two weeks?

[Yes=1; No=2; Do not know=3]

1. If yes can you name them?
   1. Milk=1
   2. Yoghurt=2
   3. Butter=3
   4. Cheese=4
   5. Cream=5
   6. Ice cream=6
   7. Other=7

Note: Circle the given answer and also put the code in the box

1. Did you (your child) eat kai (mussells) in the last few weeks?

[Yes=1; No=2; Do not know=3]

1. Did you (your child) eat lolo (squeezed coconut milk) in the last two weeks?

[Yes=1; No=2; Do not know=3]

1. Did you (your child) attend any mass gathering in the last two weeks? [Yes=1;No=2]
2. If yes name them and date please
   1. Wedding:

DD MM YYYY

- 1. Funeral:

DD MM YYYY

- 1. Religious festival:

DD MM YYYY

- 1. Other:

DD MM YYYY

**Sanitation information**

1. Do you (your child) have a toilet in the household? [Yes=1; No=2]
2. What kind of toilet facility do you (your child) have in the household
   1. Flush toilet=1
   2. Pour flush toilet=2
   3. Ventilated improved pit (VIP) latrine=3
   4. Traditional pit latrine=4
   5. Improved water seal toilet=5
   6. No facility: Bush/field/ground/stream/open sewer=6
   7. Other=7

Note: Circle the given answer and also put the code in the box

1. Have you or someone from your family has built the toilet for your use?

[Yes=1; No=2]

If “NO” go to Q65 otherwise continue

1. If yes, what kind of substrate is used to build the toilet?
   1. Clay=1
   2. Loam=2
   3. Sand=3
   4. Gravel=4
   5. Coral rubble=5
   6. Soap stone=6
   7. Other=7
   8. Do not know=8

Note: Circle the given answer and also put the code in the box

1. Do you know how deep the toilet is?
   1. Arm’s length=1
   2. Outstretched arm’s length=2
   3. More than two arm’s length=3
   4. Do not know=4
2. If you have a flush toilet please can you tell us the type of septic tank that the toilet has?

[Please check the response in Q61 to answer this question]

- 1. Concrete=1
  2. 44 gallon drum=2
  3. Plastic=3
  4. Fibre glass=4
  5. Other=5
  6. Do not know=6
  7. Do not have a flush toilet=7

Note: Circle the given answer and also put the code in the box

Ask Q 66 (to the primary caregiver) if the case or control is very young and do not use a toilet by him/her self

1. Where do you usually dispose of your child’s faeces?
   1. Toilet=1
   2. Bury=2
   3. Scatter in the yard=3
   4. Bush/ground/stream/open sewer=4
   5. Do nothing=5
   6. Other=6

Note: Circle the given answer and also put the code in the box

1. When do you (your child) usually wash your hand? [always=1; sometimes=2; never=3; not applicable=4] Check all that apply

67a. Before eating:

67b. Before cooking:

67c. Before preparing child’s food:

67d. After cleaning a child who defecated:

67e. After you defecate:

67f. Other:

1. Do you have separate source of water for hand washing? [Yes=1; No=2]
2. Do you use any disinfectant while washing hand? [Yes=1; No=2]
3. If yes what do you usually use
   1. Soap=1
   2. Ashes=2
   3. Mud=3
   4. Other___________________________=4

Note: Circle the given answer and also put the code in the box

1. Where do you usually bathe?
2. In house, piped water=1
3. In house, stored water=2
4. In river/stream=3
5. Other_________________=4

Note: Circle the given answer and also put the code in the box

1. Where do you usually wash clothes?
2. In house, piped water=1
3. In house, stored water=2
4. In river/stream=3
5. Other_________________=4

Note: Circle the given answer and also put the code in the box

1. Where do you usually prepare food?
2. In the house=1
3. In a communal outdoor setting=2
4. At the farm=3

Note: Circle the given answer and also put the code in the box

1. How often do you visit the local river/stream?
2. Daily=1
3. Weekly=2
4. Monthly=3
5. Rarely=4
6. Never=5

**Environment**

1. How has the rainfall been in the last two weeks?
2. Heavy rain=1
3. Moderate rain=2
4. Little rain=3
5. No rain=4
6. How has the rainfall been in the last two months but prior to last two weeks?
7. Heavy rain=1
8. Moderate rain=2
9. Little rain=3
10. No rain=4
11. Have you been affected by a cyclone/tropical storm in the last 2 weeks?

[Yes=1; No=2]

1. Have you been affected by a cyclone/tropical storm in the last 2 months?

[Yes=1; No=2]

1. Have you been evacuated from your home in the last two weeks?

[Yes=1; No=2]

1. Have you been evacuated from your home in the last two months?

[Yes=1; No=2]

1. Have you been afffected by drought in the last two weeks?

[Yes=1; No=2]

1. Have you been affected by drought in the last 2 months?

[Yes=1; No=2]

1. Did your house flood in the last two weeks?

[Yes=1; No=2]

1. Did your house flood in the last two months?

[Yes=1; No=2]

1. Has there been flooding adjacent to your house in the last two weeks?

[Yes=1; No=2]

1. Has there been flooding adjacent to your house in the last two months?

[Yes=1; No=2]

1. Has the village flooded in the last two weeks?

[Yes=1; No=2]

1. Has the village flooded in the last two months?

[Yes=1; No=2]

1. Has the toilet/latrine that you use been flooded in the last 2 weeks?

[Yes=1; No=2]

1. Has the toilet/latrine that you use been flooded in the last 2 months?

[Yes=1; No=2]

1. Has the nearest stream/river flooded in the last two weeks?

[Yes=1; No=2]

1. Has the nearest stream/river flooded in the last two months?

[Yes=1; No=2]

1. Are there farms above where water is collected for household use?

[Yes=1; No=2]

1. Are there livestock above where water is collected for household use?

[Yes=1; No=2]

1. Are there latrines/toilets above where water is collected for household use?

[Yes=1; No=2]

1. Is there logging activities higher in the river basin?

[Yes=1; No=2]

1. Are there road building activities higher in the river basin?

[Yes=1; No=2]

1. Are there mining activities (including sand dredging) higher in the river basin?

[Yes=1; No=2]

1. Are there dams higher in the river basin?

[Yes=1; No=2]

1. Do you use the nearby river/stream for fishing or collecting food

[Yes=1; No=2]

If yes to Q 100 then continue otherwise stop interview here

101.If so, what are you fishing/collecting?

a. Fish (Ika) [Yes=1; No=2]

b. Clam (Kai) [Yes=1; No=2]

c. Prawns (Ura) [Yes=1; No=2]

d. Other_______ [Yes=1; No=2]

Note: Circle the given answer and also put the code in the box

102. If you go for fishing how much time do you spend per trip in the river?

a. 0-1 hr [Yes=1; No=2]

b.1-3 hrs [Yes=1; No=2]

c. Half a day [Yes=1; No=2]

d. One full day [Yes=1; No=2]

e.Other____________ [Yes=1; No=2]

Note: Circle the given answer and also put the code in the box

Initials of the interviewer:

Signature of the interviewer:___________________________________

Date:
