# Supplementary material for: Epidemiology and risk factors for typhoid fever in Central Division, Fiji, 2014–2017: A case-control study
Source: PLoS Negl Trop Dis. 2018 Jun 8;12(6):e0006571. doi: 10.1371/journal.pntd.0006571 (PMC6010302; doi:10.1371/journal.pntd.0006571)
Supplement: S1 Fig — (DOCX) [file pntd.0006571.s004.docx]

**S1 Fig: Directed acyclic graph used to guide variable selection**

**
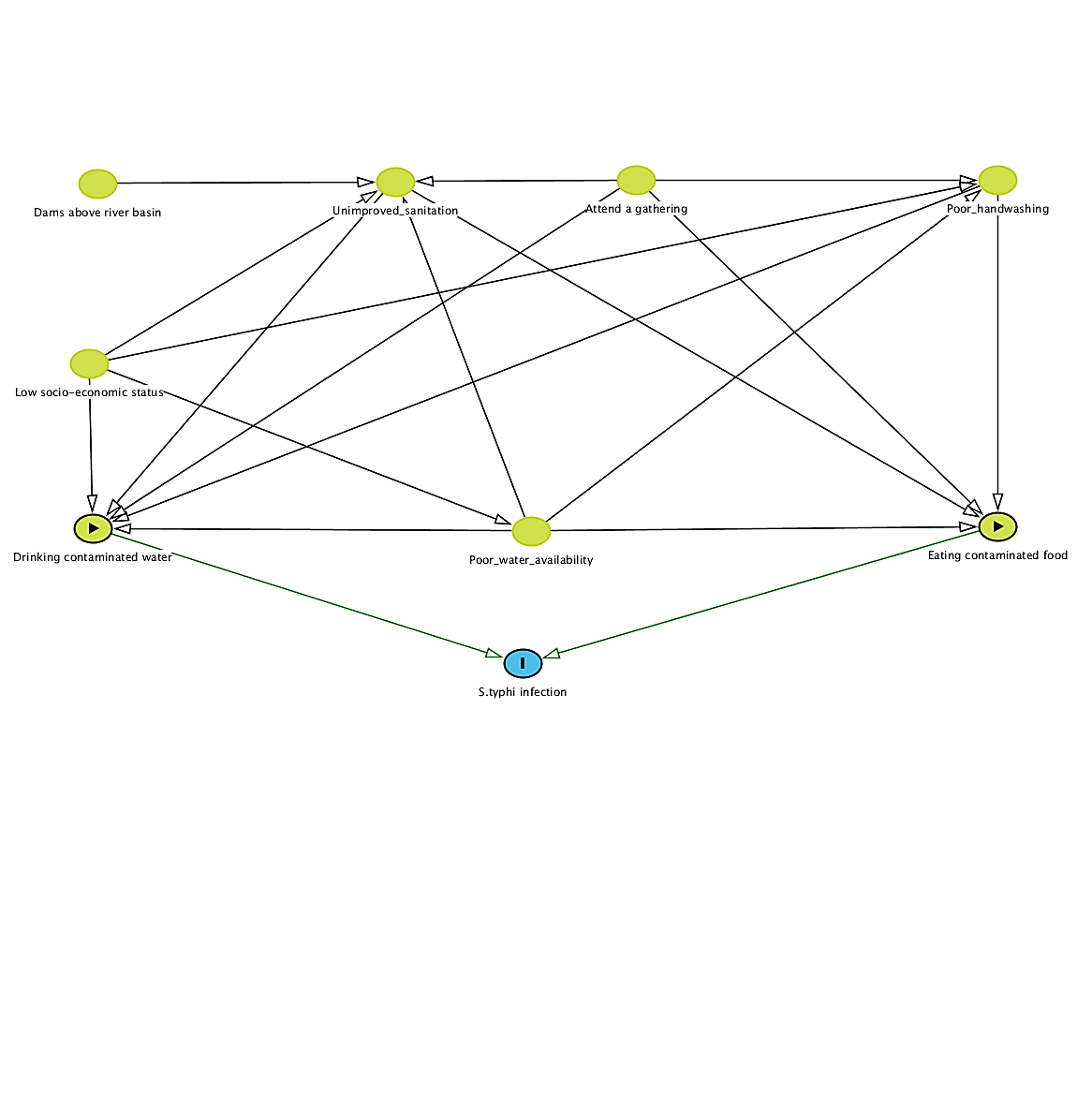
**

Directed acyclic graph was created using DAGitty, a browser based software.

Johannes Textor, Benito van der Zander, Mark K. Gilthorpe, Maciej Liskiewicz, George T.H. Ellison. 
[Robust causal inference using directed acyclic graphs: the R package 'dagitty'.](http://dx.doi.org/10.1093/ije/dyw341)
*International Journal of Epidemiology* 45(6):1887-1894, 2016.
