# Supplementary material for: Epidemiology and risk factors for typhoid fever in Central Division, Fiji, 2014–2017: A case-control study
Source: PLoS Negl Trop Dis. 2018 Jun 8;12(6):e0006571. doi: 10.1371/journal.pntd.0006571 (PMC6010302; doi:10.1371/journal.pntd.0006571)
Supplement: S1 Table — (DOCX) [file pntd.0006571.s005.docx]

**S1 Table : Univariable analysis of risk factors for blood-culture confirmed *Salmonella* Typhi among 175 cases and 175 age, ethnicity, and near-neighborhood matched controls, Central Division, Fiji, 2014 – 2017**

| Risk factor/ Exposure | Number and (%) of cases with risk factor/ exposure  Total no. of cases = 175 | | Number and (%) of controls with risk factor/  exposure  Total no. of controls = 174 | | Conditional odds ratio | Exact 95% confidence Intervals | p-value |
| --- | --- | --- | --- | --- | --- | --- | --- |
|  | N | (%) | N | (%) |  |  |  |
| **Household** |  |  |  |  |  |  |  |
| High socio-economic status index | 32 | (18.3) | 39 | (22.4) | ref |  |  |
| Medium socio-economic status index | 57 | (32.6) | 71 | (40.8) | 1.16 | 0.58-2.32 | 0.674 |
| Low socio-economic status index | 86 | (49.1) | 64 | (36.8) | 2.03 | 0.98-4.20 | 0.056 |
| Animals on household | 86 | (49.1) | 106 | (60.9) | 0.50 | 0.29-0.83 | 0.008 |
| **Water source, treatment, and drinking** |  |  |  |  |  |  |  |
| Main household water source |  |  |  |  |  |  |  |
| Piped treated | 88 | (50.3) | 86 | (49.4) | ref |  |  |
| Piped untreated | 31 | (17.7) | 36 | (20.7) | 0.42 | 0.11-1.63 | 0.211 |
| Rain water | 4 | (2.3) | 3 | (1.7) | 1.28 | 0.17-9.55 | 0.808 |
| Surface water | 52 | (29.7) | 49 | (28.2) | 1.00 | 0.18-5.47 | 1.000 |
| Main water source accessed from outside house | 118 | (67.4) | 62 | (35.6) | 2.20 | 0.76-6.33 | 0.144 |
| Water not always available from main source | 49 | (28.0) | 42 | (24.1) | 1.39 | 0.75-2.54 | 0.288 |
| Treated water in house | 50 | (28.6) | 50 | (28.7) | 0.97 | 0.57-1.62 | 0.895 |
| Stored water in house | 139 | (79.4) | 138 | (79.3) | 0.96 | 0.54-1.69 | 0.884 |
| Drank untreated water | 76 | (43.4) | 63 | (36.2) | 1.86 | 0.96-3.55 | 0.062 |
| Only drank water from main household water source | 104 | (59.4) | 165 | (94.8) | ref | - |  |
| Drank from an alternate water source (non-surface water source) | 53 | (30.3) | 7 | (4.0) | 1.21 | 0.68-2.18 | 0.520 |
| Drank from an alternate water source (surface water source) | 18 | (10.3) | 2 | (1.1) | 2.13 | 0.84-5.42 | 0.110 |
| Drank water at a mass gathering | 14 | (8.0) |  | (0.0) | 1.59 | 0.79-3.20 | 0.192 |
| Consumed ice | 69 | (39.4) | 66 | (37.9) | 1.12 | 0.70-1.77 | 0.638 |
| Drank water/other drink from a street vendor | 57 | (32.6) | 53 | (30.5) | 1.07 | 0.64-1.75 | 0.800 |
| Drank kava ^a^ | 71 | (40.6) | 82 | (47.1) | 0.68 | 0.38-1.17 | 0.168 |
| **Food & Behavior** |  |  |  |  |  |  |  |
| Did not wash produce before eating | 53 | (30.3) | 28 | (16.1) | 2.71 | 1.47-5.00 | 0.001 |
| Stored food | 121 | (69.1) | 125 | (71.8) | 0.85 | 0.51-1.40 | 0.523 |
| Shared food on the same plate | 20 | (11.4) | 21 | (12.1) | 2.43 | 1.30-4.52 | 0.005 |
| Ate outside of house | 77 | (44.0) | 52 | (29.9) | 2.10 | 1.24-3.52 | 0.005 |
| Consumed dairy products | 156 | (89.1) | 157 | (90.2) | 0.86 | 0.39-1.85 | 0.695 |
| Ate kai/mussels | 72 | (41.1) | 91 | (52.3) | 0.55 | 0.32-0.91 | 0.020 |
| Ate lolo/coconut milk | 129 | (73.7) | 137 | (78.7) | 0.64 | 0.34-1.19 | 0.163 |
| Attended a mass gathering | 64 | (36.6) | 42 | (24.1) | 2.00 | 1.18-3.37 | 0.009 |
| **Sanitation and hygiene** |  |  |  |  |  |  |  |
| Shared toilet with non-household members | 24 | (13.7) | 20 | (11.5) | 1.40 | 0.62-3.15 | 0.416 |
| Householders built their own toilet | 92 | (52.6) | 75 | (43.1) | 1.82 | 1.08-3.05 | 0.024 |
| Have a unimproved/damaged improved sewerage system ^b^ | 167 | (95.4) | 11 | (6.3) | 4.40 | 1.66-11.6 | 0.003 |
| Undamaged improved, municipal sewerage | 8 | (4.6) | 16 | (9.2) | ref | - |  |
| Unimproved pit latrine | 16 | (9.1) | 6 | (3.4) | 22.38 | 3.25-154.21 | 0.002 |
| No toilet/open defecation | 5 | (2.9) | 1 | (0.6) | 15.22 | 1.32-175.09 | 0.029 |
| Damaged improved, municipal sewerage | 7 | (4.0) | 4 | (2.3) | 7.54 | 1.28-44.45 | 0.026 |
| Improved pit latrine | 62 | (35.4) | 60 | (34.5) | 3.85 | 1.07-13.89 | 0.040 |
| Intact septic | 77 | (44.0) | 87 | (50.0) | 2.67 | 0.85-8.38 | 0.093 |
| Separate water source for washing hands | 35 | (20.0) | 41 | (23.4) | 0.76 | 0.42-1.38 | 0.369 |
| High hand washing frequency after defecation |  |  |  |  | 0.57 | 0.43-0.75 | 0.000 |
| Used soap for hand washing | 64 | (36.6) | 92 | (52.6) | 0.37 | 0.20-0.66 | 0.001 |
| **Environment** |  |  |  |  |  |  |  |
| Heavy to moderate rain 2 weeks | 87 | (49.7) | 83 | (47.7) | 1.15 | 0.68-1.95 | 0.593 |
| Heavy to moderate rain 2 months | 98 | (56.0) | 95 | (54.6) | 1.15 | 0.68-1.95 | 0.593 |
| Household evacuated 2 weeks | 2 | (1.1) | 1 | (0.6) | 2.00 | 0.18-22.0 | 0.571 |
| Household evacuated 2 months | 3 | (1.7) | 4 | (2.3) | - | - | - |
| Drought 2 weeks | 1 | (0.6) | 0 | (0.0) | - | - | - |
| Drought 2 months | 1 | (0.6) | 2 | (1.1) | - | - | - |
| Flooding adjacent 2 weeks | 5 | (2.9) | 5 | (2.9) | 1.00 | 0.20-4.95 | 1.000 |
| Flooding adjacent 2 months | 2 | (1.1) | 5 | (2.9) | 0.25 | 0.02-2.23 | 0.215 |
| Village flooded 2 weeks | 6 | (3.4) | 4 | (2.3) | 2.00 | 0.36-10.9 | 0.423 |
| Village flooded 2 months | 5 | (2.9) | 6 | (3.4) | 0.75 | 0.16-3.35 | 0.706 |
| Toilet flooded 2 weeks | 2 | (1.1) | 0 | (0.0) | - | - | - |
| Toilet flooded 2 months | 1 | (0.6) | 1 | (0.6) | 1.00 | 0.06-15.9 | 1.000 |
| River/stream flooded 2 weeks | 14 | (8.0) | 9 | (5.2) | 2.00 | 0.60-6.64 | 0.258 |
| River/stream flooded 2 months | 19 | (10.9) | 11 | (6.3) | 2.00 | 0.80-4.95 | 0.134 |
| Farms above water collection | 11 | (6.3) | 7 | (4.0) | 2.33 | 0.60-9.02 | 0.220 |
| Livestock above water collection | 7 | (4.0) | 3 | (1.7) | 5.00 | 0.58-42.7 | 0.142 |
| Logging above river basin | 1 | (0.6) | 2 | (1.1) | - | - | - |
| Road building above river basin | 3 | (1.7) | 6 | (3.4) | 0.25 | 0.02-2.23 | 0.215 |
| Dams above river basin | 71 | (40.6) | 59 | (33.9) | 1.83 | 0.90-3.70 | 0.091 |

Odds ratios were estimated using conditional logistic regression. All exposures are focused on the 2-week period prior to onset of symptoms for cases and the date of recruitment for controls, unless specified otherwise.

^a^ Traditional Fijian drink (*Piper methysticum*).

^b^ Summary variable of all sanitation facilities.
