# Supplementary material for: Epidemiology and risk factors for typhoid fever in Central Division, Fiji, 2014–2017: A case-control study
Source: PLoS Negl Trop Dis. 2018 Jun 8;12(6):e0006571. doi: 10.1371/journal.pntd.0006571 (PMC6010302; doi:10.1371/journal.pntd.0006571)
Supplement: S2 Table — (DOCX) [file pntd.0006571.s006.docx]

**S2 Table: Univariable analysis of risk factors for blood-culture confirmed *Salmonella* Typhi among 175 cases and 175 age, ethnicity, and distant-neighborhood matched controls, Central Division, Fiji, 2014 – 2017**

| Risk factor/ Exposure | Number and (%) of cases with risk factor/ exposure  Total no. of cases = 175 | | Number and (%) of controls with risk factor/  exposure  Total no. of controls = 175 | | Conditional odds ratio | Exact 95% confidence Intervals | p-value |
| --- | --- | --- | --- | --- | --- | --- | --- |
|  | N | (%) | N | (%) |  |  |  |
| **Household** |  |  |  |  |  |  |  |
| High socio-economic status index | 32 | (18.3) | 59 | (33.7) | ref |  |  |
| Medium socio-economic status index | 57 | (32.6) | 56 | (32.0) | 2.46 | 1.26-4.79 | 0.008 |
| Low socio-economic status index | 86 | (49.1) | 60 | (34.3) | 4.10 | 2.02-8.35 | 0.000 |
| Animals on household | 86 | (49.1) | 88 | (50.3) | 0.93 | 0.60-1.42 | 0.745 |
| **Water source, treatment, and drinking** |  |  |  |  |  |  |  |
| Main household water source |  |  |  |  |  |  |  |
| Piped treated | 88 | (50.3) | 85 | (48.6) | ref |  |  |
| Piped untreated | 31 | (17.7) | 42 | (24.0) | 0.48 | 0.15-1.49 | 0.204 |
| Rain water | 4 | (2.3) | 2 | (1.1) | 1.79 | 0.32-9.97 | 0.505 |
| Surface water | 52 | (29.7) | 46 | (26.3) | 1.09 | 0.28-4.22 | 0.902 |
| Main water source accessed from outside house | 118 | (67.4) | 56 | (32.0) | 2.71 | 1.14-6.45 | 0.024 |
| Water not always available from main source | 49 | (28.0) | 20 | (11.4) | 4.22 | 2.04-8.73 | 0.000 |
| Treated water in house | 50 | (28.6) | 57 | (32.6) | 0.74 | 0.44-1.23 | 0.251 |
| Stored water in house | 139 | (79.4) | 130 | (74.3) | 1.36 | 0.81-2.27 | 0.243 |
| Drank untreated water | 76 | (43.4) | 62 | (35.4) | 1.82 | 1.00-3.29 | 0.047 |
| Only drank water from main household water source | 104 | (59.4) | 166 | (94.9) | ref | - |  |
| Drank from an alternate water source (non-surface water source) | 53 | (30.3) | 7 | (4.0) | 1.22 | 0.70-2.14 | 0.483 |
| Drank from an alternate water source (surface water source) | 18 | (10.3) | 2 | (1.1) | 4.84 | 1.38-16.95 | 0.014 |
| Drank water at a mass gathering | 14 | (8.0) | 9 | (5.1) | 1.38 | 0.75-2.51 | 0.302 |
| Consumed ice | 69 | (39.4) | 65 | (37.1) | 1.14 | 0.72-1.78 | 0.569 |
| Drank water/other drink from a street vendor | 57 | (32.6) | 44 | (25.1) | 1.54 | 0.92-2.57 | 0.099 |
| Drank kava ^a^ | 71 | (40.6) | 83 | (47.4) | 0.63 | 0.35-1.09 | 0.099 |
| **Food & Behavior** |  |  |  |  |  |  |  |
| Did not wash produce before eating | 53 | (30.3) | 20 | (11.4) | 4.00 | 2.06-7.74 | 0.000 |
| Stored food | 121 | (69.1) | 127 | (72.6) | 0.80 | 0.46-1.36 | 0.415 |
| Shared food on the same plate | 20 | (11.4) | 26 | (14.9) | 1.76 | 0.97-3.19 | 0.061 |
| Ate outside of house | 77 | (44.0) | 69 | (39.4) | 1.22 | 0.78-1.87 | 0.378 |
| Consumed dairy products | 156 | (89.1) | 161 | (92.0) | 0.67 | 0.29-1.48 | 0.321 |
| Ate kai/mussels | 72 | (41.1) | 90 | (51.4) | 0.62 | 0.38-0.98 | 0.041 |
| Ate lolo/coconut milk | 129 | (73.7) | 140 | (80.0) | 0.60 | 0.31-1.13 | 0.118 |
| Attended a mass gathering | 64 | (36.6) | 54 | (30.9) | 1.29 | 0.82-1.99 | 0.265 |
| **Sanitation and hygiene** |  |  |  |  |  |  |  |
| Shared toilet with non-household members | 24 | (13.7) | 22 | (12.6) | 1.17 | 0.53-2.52 | 0.695 |
| Householders built their own toilet | 92 | (52.6) | 78 | (44.6) | 1.56 | 0.95-2.52 | 0.073 |
| Have a unimproved/damaged improved sewerage system ^b^ | 167 | (95.4) | 12 | (6.9) | 3.67 | 1.48-9.04 | 0.005 |
| Undamaged improved, municipal sewerage | 8 | (4.6) | 14 | (8.0) | ref | - |  |
| Unimproved pit latrine | 16 | (9.1) | 4 | (2.3) | 38.62 | 3.83-389.09 | 0.002 |
| No toilet/open defecation | 5 | (2.9) | 2 | (1.1) | 13.44 | 1.14-158.87 | 0.039 |
| Damaged improved, municipal sewerage | 7 | (4.0) | 6 | (3.4) | 3.75 | 0.73-19.43 | 0.115 |
| Improved pit latrine | 62 | (35.4) | 50 | (28.6) | 4.36 | 1.37-13.90 | 0.013 |
| Intact septic | 77 | (44.0) | 99 | (56.6) | 2.06 | 0.71-5.92 | 0.181 |
| Separate water source for washing hands | 35 | (20.0) | 48 | (27.4) | 0.56 | 0.32-1.00 | 0.052 |
| High hand washing frequency after defecation |  |  |  |  | 0.55 | 0.42-0.72 | 0.000 |
| Used soap for hand washing | 64 | (36.6) | 100 | (57.1) | 0.33 | 0.19-0.58 | 0.000 |
| **Environment** |  |  |  |  |  |  |  |
| Heavy to moderate rain 2 weeks | 87 | (49.7) | 86 | (49.1) | 1.03 | 0.63-1.68 | 0.901 |
| Heavy to moderate rain 2 months | 98 | (56.0) | 86 | (49.1) | 1.54 | 0.92-2.58 | 0.099 |
| Household evacuated 2 weeks | 2 | (1.1) | 5 | (2.9) | 0.25 | 0.02-2.23 | 0.215 |
| Household evacuated 2 months | 3 | (1.7) | 3 | (1.7) | 1.00 | 0.06-15.9 | 1.000 |
| Drought 2 weeks | 1 | (0.6) | 1 | (0.6) | 1.00 | 0.06-15.9 | 1.000 |
| Drought 2 months | 1 | (0.6) | 1 | (0.6) | 1.00 | 0.06-15.9 | 1.000 |
| Flooding adjacent 2 weeks | 5 | (2.9) | 7 | (4.0) | 0.67 | 0.18-2.36 | 0.530 |
| Flooding adjacent 2 months | 2 | (1.1) | 3 | (1.7) | 0.67 | 0.11-3.98 | 0.657 |
| Village flooded 2 weeks | 6 | (3.4) | 3 | (1.7) | 2.00 | 0.50-7.99 | 0.327 |
| Village flooded 2 months | 5 | (2.9) | 2 | (1.1) | 2.50 | 0.48-12.8 | 0.273 |
| Toilet flooded 2 weeks | 2 | (1.1) | 1 | (0.6) | 2.00 | 0.18-22.0 | 0.571 |
| Toilet flooded 2 months | 1 | (0.6) | 0 | (0.0) | - | - | - |
| River/stream flooded 2 weeks | 14 | (8.0) | 7 | (4.0) | 2.17 | 0.82-5.70 | 0.117 |
| River/stream flooded 2 months | 19 | (10.9) | 8 | (4.6) | 2.83 | 1.11-7.18 | 0.028 |
| Farms above water collection | 11 | (6.3) | 6 | (3.4) | 2.67 | 0.70-10.0 | 0.147 |
| Livestock above water collection | 7 | (4.0) | 2 | (1.1) | 3.50 | 0.72-16.8 | 0.118 |
| Logging above river basin | 1 | (0.6) | 2 | (1.1) | 0.50 | 0.04-5.51 | 0.571 |
| Road building above river basin | 3 | (1.7) | 5 | (2.9) | - | - | - |
| Dams above river basin | 71 | (40.6) | 53 | (30.3) | 2.38 | 1.24-4.55 | 0.009 |

Odds ratios were estimated using conditional logistic regression. All exposures are focused on the 2-week period prior to onset of symptoms for cases and the date of recruitment for controls, unless specified otherwise.

^a^ Traditional Fijian drink (*Piper methysticum*).

^b^ Summary variable of all sanitation facilities.
